# Exploring the Sulfatase 1 Catch Bond Free Energy Landscape using Jarzynski’s Equality

**DOI:** 10.1038/s41598-018-35120-0

**Published:** 2018-11-15

**Authors:** Volker Walhorn, Ann-Kristin Möller, Christian Bartz, Thomas Dierks, Dario Anselmetti

**Affiliations:** 1 Experimental Biophysics and Applied Nanoscience, Faculty of Physics, Bielefeld University, Bielefeld, Germany; 2 Biochemistry I, Faculty of Chemistry, Bielefeld University, Bielefeld, Germany

## Abstract

In non-covalent biological adhesion, molecular bonds commonly exhibit a monotonously decreasing life time when subjected to tensile forces (slip bonds). In contrast, catch bonds behave counter intuitively, as they show an increased life time within a certain force interval. To date only a hand full of catch bond displaying systems have been identified. In order to unveil their nature, a number of structural and phenomenological models have been introduced. Regardless of the individual causes for catch bond behavior, it appears evident that the free energy landscapes of these interactions bear more than one binding state. Here, we investigated the catch bond interaction between the hydrophilic domain of the human cell surface sulfatase 1 (Sulf1HD) and its physiological substrate heparan sulfate (HS) by atomic force microscopy based single molecule force spectroscopy (AFM-SMFS). Using Jarzynski’s equality, we estimated the associated Gibbs free energy and provide a comprehensive thermodynamic and kinetic characterization of Sulf1HD/HS interaction. Interestingly, the binding potential landscape exhibits two distinct potential wells which confirms the recently suggested two state binding. Even though structural data of Sulf1HD is lacking, our results allow to draft a detailed picture of the directed and processive desulfation of HS.

## Introduction

**Figure 1:**
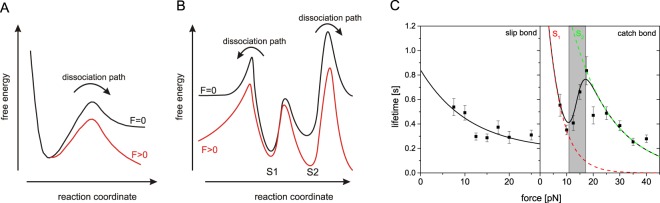
Flat representation of free energy landscapes of slip and catch bonds, respectively. Here, the reaction coordinate is the path along which the system evolves under the impact of an external force. In the experiment this is measured as the molecular extension. (**A**) Exemplary single well free energy landscape of a molecular slip bond. The black curve depicts the binding potential of a molecular complex without external force. In contrast, the potential is distorted (lowering of the transition state) when the complex is subjected to an external force (red curve). (**B**) Double well free energy landscape as proposed for catch bond interaction. The occupancy of the states *S*1 and *S*2 governed by equilibrium thermodynamics. Without an external force (black plot) solely *S*1 is occupied. By increasing the force *S*2 is successively populated. Within the transition (catch) regime both *S*1 and *S*2 are populated. The estimated complex life time therefore is a superposition of the individual life time of both states. (**C**) Characteristic force life time plots of a slip and catch bond, respectively. The plots exhibit the complex life time of Sulf1HD bound to heparosan N-sulfate lacking 6-O-sulfates (left panel) and Sulf1HD’s physiological substrate HS (right panel)^22^. The catch regime (gray) marks the transition between two individual slip bond states *S*1 (dashed red) and *S*2 (dashed green).

Molecular recognition is involved in many biological processes such as (cell) adhesion, catalysis, signal transduction and allosteric regulation. These non-covalent interactions establish short-lived but highly specific bonds between distinct molecules. Conceptually, these interactions can be divided into slip- and catch bonds, respectively. Slip bonds exhibit a monotonously decreasing bond life time under an increasing mechanical load, whereas catch bonds bind tighter within a certain force interval. Originally slip bonds were introduced by Bell in 1978 who quantified their kinetics on the basis of reaction rate theory^1^. To date, the nature of slip bonds is well-understood. Their dissociation can be rationalized by a path in a single-well energy landscape that leads from a bound state across an activation energy barrier (Fig. 1A). Since the first slip bond-like dissociation rate constant was reported in 1995, this concept was proven many times^2–9^. In contrast, the non intuitive behavior of catch bonds is still a matter of debate. Originally postulated by Dembo and coworkers in 1988, the first evidence of catch bond-like dissociation was not found until 2003^10^. Since then only a few systems exhibiting catch bond-like dissociation have been identified^11–14^.

In order to unveil the underlying principles a number of phenomenological models have been developed^15–20^. Even though it is not clear whether the origin of catch bond behavior in the diverse biological systems can be attributed to a single cause, their unintuitive dissociation behavior cannot be rationalized by a trivial single-well energy landscape exhibiting a single dissociation path. The two state two path model approximates all data published so far reasonably well^21^. According to this model, the molecular complex can bind in two different states *S*1 and *S*2 that are separated by an energy barrier (Fig. 1B). In case a single force extension experiment is done at time scales that are significantly slower than molecular relaxation, the occupancy of both states can be estimated by equilibrium thermodynamics. Generally, the low force state *S*1 is populated at forces below a certain threshold force *F*_*min*_. Increasing the load on the molecular complex results in slip-like dissociation until the applied force exceeds *F*_*min*_. Then, *S*1 is successively depopulated while the population of the high force state *S*2 rises. Within this transition regime the observed average complex life time is the population-weighed life time of both states^22^. Consequently, the observed life time increases until *S*1 is depleted. Further increasing the external force again leads to slip bond-like dissociation. Within the two state two path model catch bond dissociation is regarded as the force induced transition from a brittle low force state (*S*1) to a tighter bound high force state (*S*2). Interestingly, not all catch bond systems exhibit this tri-phasic (slip-catch-slip) behavior. P-selectin and FimH for example dissociate in a catch-slip fashion^10,23^. To date it is not clear, if an initial slip regime does exist or if it is not detectable due to limited force sensitivity. However, the two state two path model also covers systems of catch-slip type. Here, the low force (catch) binding mode can be regarded as a superposition of two states. It therefore appears evident, that catch bonds are inherently linked to non-trivial free energy landscapes.

**Figure 2:**
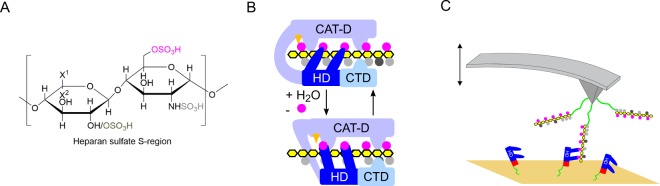
(**A**) Typical disaccharide units of heparan sulfate S-regions. Disaccharide units of highly sulfated heparan sulfate S-regions consist of an uronic acid (either iduronic acid: X^1^ = H and X^2^ = COOH or glucuronic acid: X^1^ = COOH and X^2^ = H, with or without 2-O-sulfation (dark grey)) 1-4 linked to a modified glucosamine. Up to 8 consecutive disaccharide units were shown to have the depicted structure, where N-sulfation (light grey) is uniform and 6-O-sulfation (magenta) of the glucosamine residue is predominant^45^. Regions of heparan sulfate with di- or tri-sulfated disaccharide units are the primary substrate of Sulf1^44^ and 6-O sulfation is required for binding by the hydrophilic domain of Sulf1. (**B**) Hypothetical model of heparan sulfate 6-O-desulfation by Sulf1. The hydrophilic domain (HD) of Sulf1 (dark blue) binds to 6-O-sulfated sites (magenta) in highly sulfated S-regions of heparan sulfate (HS, yellow). Additional contacts between heparan sulfate and the catalytic domain (CAT-D) and the C-terminal domain (CTD) of Sulf1 may be formed. The 6-O-desulfation at the active site of Sulf1 (orange) could induce a shift to a tensed state in which HD and HS are interacting with high affinity. Although structural details are unknown, an associated conformational change could potentially facilitate progression of Sulf1 to the next 6-O-sulfated residue within highly sulfated regions of HS. N-sulfate groups (light grey dots) and 2-O-sulfate groups (dark grey dots) of HS are indicated. (**C**) Typical AFM setup to probe the dissociation forces of molecular complexes. HS polymers (yellow, 6-O-sulfates magenta circles) are covalently immobilized to a gold AFM tip via PEG-NHS ester disulfite linkers (green). MBP-HD fusion proteins (HD blue, MBP red) are bound to the gold substrates using the same linker.

In this work, we focus on the previously reported catch bond behavior of the hydrophilic domain (HD) of the human cell surface sulfatase 1 (Sulf1) and its physiological substrate heparan sulfate (HS)^22^ (Fig. 2A). In constant force experiments Sulf1HD exhibited a tri-phasic slip-catch-slip type of dissociation with a catch regime between approx. 10–17 pN (Fig. 1C right panel). Bimodal dissociation force distributions obtained in force ramp experiments at several constant pulling velocities suggest a free energy landscape that bears two different, well-defined binding states. Control experiments with glycosaminoglycans (GAG) lacking 6-O-sulfates clearly exhibited dissociation of slip type (Fig. 1C left panel). In order to further prove this hypothesis and to model the free energy landscape of this interaction, we used Jarzynski’s equality which allowed us to estimate the equilibrium Gibbs free energy of a bound state from a series of non-equilibrium experiments. Consistent with recent results, we identified two binding states that differ by a free energy of approx. 20 kJmol^−1^. We furthermore provide a comprehensive thermodynamic and kinetic characterization of Sulf1HD/HS binding which leads to a refined picture of the directed and processive desulfation of HS. As a result, it appears reasonable that allosteric regulation steers both catalytic HS desulfation and the processive motion of Sulf1 along the HS polysaccharide chain (Fig. 2B).

## Results and Discussion

**Figure 3:**
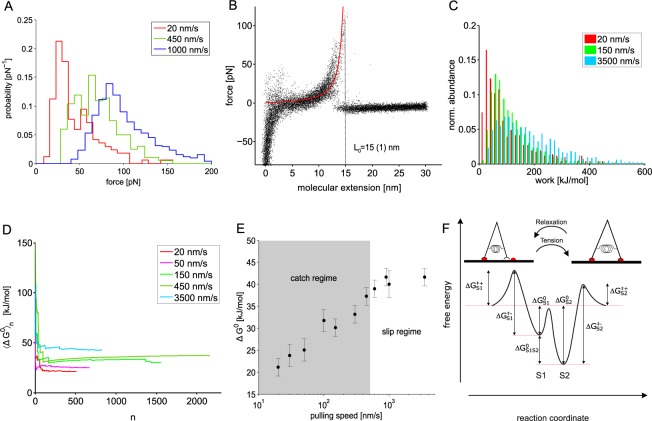
(**A**) Dissociation force histograms acquired at different pulling speeds. The histograms evolve from double-peaked distributions at low and medium velocities to single peaked distributions. The occurrence and height of the peaks corresponds to the occupancy of the individual states *S*1 and *S*2. At a pulling velocity of 1000 nm s^-1^ (blue plot) the low force peak referring to dissociation from *S*1 vanished completely^22^. (**B**) A set (*n* = 50) of molecular force extension curves approximated to a worm like chain curve (solid red) *l*_*p*_ = 0.4(0.1) nm and *L*_0_ = 15(1) nm. (**C**) Work distributions for different velocities. (**D**) Evolution of the Jarzynski equality. The exponentially averaged work *W*_*n*_ plotted versus the number of dissociation events *n*. (**E**) Estimated differences in free energy Δ*G*^0^ at various pulling velocities. Within the catch (transition) regime (gray) *S*1 is successively depleted whereas *S*2 becomes continuously populated. (**F**) Proposed free energy landscape (not to scale) for Sulf1HD/HS interaction. Here, the low force (*S*1) and high force binding state (*S*2) are shown in one potential landscape. Depending on the conformational state Sulf1HD could either bind in the low or high force state. In the mechanically relaxed state Sulf1HD binds only partially to the HS substrate (*S*1). When subjected to an external force Sulf1HD is stretched such that it can cross the transition state and establish additional bonds to the HS strand (*S*2).

In order to analyze and quantify the binding behaviour of Sulf1HD towards its physiological substrate HS, we covalently linked Sulf1HD and HS to a template stripped gold substrate and AFM-cantilver, respecively (Fig. 2C). More than 150,000 force extension curves were acquired in a series of single molecule force spectroscopy experiments at several constant pulling velocities ranging from 20 nm s^−1^ to 3500 nm s^−1^. We yielded dissociation force spectra that exhibited an evolution from bimodal to unimodal distributions for increasing loading rates (Fig. 3A). At speeds up to 450 nm s^−1^ we found force spectra that exhibited distinct low and high force peaks, which we attribute to individual binding states (*S*1 and *S*2). For increasing loading rates, the low force peak *S*1 degenerates and vanishes completely, whereas the high force peak *S*2 increases successively. In our previous work, we demonstrated that the evolution of the force spectra correlates with the population of corresponding states^22^. Consequently, each bimodal dissociation force distribution contains two distinct unimodal but overlapping force spectra characterizing the dissociation from the corresponding binding states. We furthermore analyzed this data within the framework of the Bell Evans Model and estimated the dissociation rate constants ${k}_{S\mathrm{1,}S2}^{-}$ and reaction lengths ${x}_{S\mathrm{1,}S2}^{\ddagger }$ for each of these states^24^. Within the scope of this work, we regard the area under the non-linear force ramp which is a measure of the work *W* that was dissipated to break the molecular complex. The Jarzynski equality allows us to link a sufficiently large set of adhesion energies *W*_*n*_ acquired in non-equilibrium experiments to the difference in the Gibbs free energy Δ*G*^0^ in thermodynamic equilibrium. Yet, *W* is biased by effects such as linker unfolding which adds to the true Δ*G*^0^. It is therefore preferential to probe a system bidirectionally (forward and backward force extension curves)^25^. This however, is not applicable to our type of experiments. It is nevertheless possible to minimize the impact of linker unfolding below 10–15% by only accepting dissociation events with a preferably short dissociation length^26^. In our experiments we therefore chose a very short linker and accepted only force extension curves for further analysis that exhibited a contour length in the range of 8 nm  to 20 nm (for details refer to Materials and Methods).

By means of evaluating the area under the extension curves we estimated the work distribution for each pulling velocity (Fig. 3C). Δ*G*^0^ then results from the evolution of 〈Δ*G*^0^〉_*n*_ (Fig. 3D). Notably, 〈Δ*G*^0^〉_*n*_ converges fast after a few hundred dissociation events thus providing a robust estimate of Δ*G*^0^ ^26^. We found that along with the evolution of the dissociation force distributions the difference in free energy increased from 21.1(20) kJmol^−1^ for the low force state *S*1 to 40.9(20) kJmol^−1^ for the high force state *S*2 (Fig. 3E,F). Of note, the latter is the average Δ*G*^0^ for pulling speeds >450 nms^−1^ (slip regime, Fig. 3E). We ascribe the increase of Δ*G*^0^ to the successive shift of the population from the brittle state *S*1 to the tighter bound state *S*2 as predicted by the two state two path model. At pulling velocities up to 450 nms^−1^ we observe a bimodal dissociation force distribution indicating dissociation events from *S*1 and *S*2, respectively. Applying the Jarzynski equality to force extension profiles within this speed range therefore leads to a superposition of both states as dissociation events from both states contribute to the estimate of Δ*G*^0^. Nevertheless, the exponential weighing by the Jarzynski equality diminishes the impact of interfering high force events and the estimated free energy difference of ${\rm{\Delta }}{G}_{S1}^{0}=21.1\pm 2$ kJmol^−1^ at a pulling speed of 20 nms^−1^ can therefore be regarded as an upper limit of the true difference in Gibbs free energy. Further reducing the pulling velocity would not result in a more accurate estimate as the dissociation events from *S*1 approach the detection limit which we set as ≈15 pN corresponding to three times the root mean square (RMS) of the force curve base line noise. Consequently, at lower speeds the set of *W*_*n*_ would lack a substantial number of low force dissociation events. At pulling velocities above 450 nms^−1^ we exclusively observe dissociation from the high force state *S*2. Accordingly, we estimated as ${\rm{\Delta }}{G}_{S2}^{0}=40.9\pm 2$ kJmol^−1^ as free energy difference of the binding state *S*2. Notably, the energy difference between both binding states of ${\rm{\Delta }}{G}_{S1S2}^{0}=19.8$ kJmol^−1^ estimated here coincides nicely with the 22 kJmol^−1^ obtained by single molecule force clamp spectroscopy experiments that were analyzed on the basis of the two state two path model^22^.

An exact estimate of Δ*G*^0^ is prerequisite to further quantify the thermodynamics and kinetics of this binding reaction and to model the corresponding free energy landscape (Fig. 3F). Using equation 4, Δ*G*^0^ is directly related to the equilibrium constant of dissociation *K* and to the association rates ${k}_{S\mathrm{1,}S2}^{+}$. We determined *K*_*S*1_ = 167(89) *μ*M for state *S*1, *K*_*S*2_ = 49(25) nM for state *S*2, respectively.

Using the dissociation rate constants ${k}_{S\mathrm{1,}S2}^{-}$, as evaluated previously, we estimated the association rates as ${k}_{S1}^{+}=299(374)\,{{\rm{M}}}^{-1}{{\rm{s}}}^{-1}$ and ${k}_{S2}^{+}=\mathrm{306(306)}\times {10}^{6}\,{{\rm{M}}}^{-1}{{\rm{s}}}^{-1}$ for *S*1 and *S*2, respectively^22^. Interestingly, the association rate constant ${k}_{S2}^{+}$ for the tight bound state *S*2 is orders of magnitude larger than association to the low affinity state *S*1. Despite the faster binding kinetics and higher affinity, we predominantly observe dissociation from the low affinity state *S*1 at low pulling velocities (Fig. 3A) which is in full agreement with the two state two path model. Apparently, *S*2 is not directly accessible from free solution. Instead binding in the low force state *S*1 and a conformational change (by an external force) seem to be prerequisite to access the tighter bound state *S*2. In an earlier study, we analyzed several fragments of Sulf1HD and identified at least two different, well-separated binding sites^27^. Therefore, we presume, that while bound in the low force state *S*1 not all binding sites associate to the HS strand. By applying a force, *S*2 becomes accessible and the interstate energy barrier can be crossed (Fig. 3F). The external stimulus also goes along with a discrete conformational transition, which drives Sulf1HD out of the mechanically relaxed state allowing to establish extra bonds to the HS strand. The low force state *S*1 accordingly refers to a loosely bound mechanically relaxed state whereas in the state *S*2 Sulf1HD is in a mechanically strained conformation but it can associate tightly to the HS substrate. Within the framework of conceptual catch bond models, there is no viable reaction path leading directly from the unbound (mechanically relaxed) to the strained bound state *S*2^19^. Hence, partial binding to the HS polysaccharide and an external stimulus are prerequisite for the conformational transition and binding in the state *S*2. The association rate constant ${k}_{S2}^{+}$ accordingly does not apply for association of the mechanically relaxed state of Sulf1HD.

Moreover, we determined the height of the activation energy barrier ${G}^{\ddagger +/-}$ (Eqs 2 and 3) of the association (+) and dissociation (−) path for each of the states yielding ${G}_{S1}^{\ddagger +}=33.9(40)\,{{\rm{kJmol}}}^{-1}$ and ${G}_{S1}^{\ddagger -}\mathrm{=55}(1)\,{{\rm{kJmol}}}^{-1}$ for the low force state *S*1 and ${G}_{S2}^{\ddagger +}\mathrm{=17.1}(30)\,{{\rm{kJmol}}}^{-1}$, ${G}_{S2}^{\ddagger -}\mathrm{=58}(1)\,{{\rm{kJmol}}}^{-1}$ for the high force state *S*2 (Fig. 3F).

Evidently, Sulf1HD plays the key role in processive HS desulfation^22,27,28^. Modelling the free energy landscapes combined with previous findings allowed us to determine its contribution to the directed processivity of catalytic HS desulfation. Interestingly, the Sulf1 catalytic domain (CAT-D) itself, when fused directly to the C-terminal domain (CTD) thus lacking HD, is sufficient for arylsulfatase activity, i.e. hydrolysis of small arylsulfate pseudosubstrates^29^. However, without HD no processive activity towards HS-derived oligosaccharides with multiple glucosamine-6-O-sulfates was observed^28^. Moreover, in control experiments with GAGs lacking 6-O-sulfates Sulf1HD did not exhibit catch bond dissociation^22^. Therefore, we consider 6-O-sulfates as allosteric effectors that mediate the transition between two different binding states. Recently, Seffouh and coworkers drafted a picture of the directed 6-O-desulfation of HS^28^. Here, Sulf1 binds to the most upstream 6-O-sulfate of a highly sulfated S-domain in such a way that it is located in vicinity of the catalytic center. After desulfation Sulf1 proceeds to the next downstream sulfate group. This process is repeated until the last 6-O-sulfate of an S-domain is hydrolyzed. Taking our findings into account, we can deduce the functionality of the full length protein and extend this picture to a detailed scheme of the molecular machinery that drives the cyclic and directed desulfation of HS. We presume that this process is realized by cyclic conformational transitions that feed 6-O-sulfates to the catalytic center and drag the sulfatase along the GAG backbone in an inchworm-like manner. This can be rationalized by a finely tuned interplay between different HS binding sites. Alternated dissociation and (re-) binding at specific loci on the HS strand combined with cyclic conformational transitions can drive the directed movement along the HS chain in a stepwise process that is coupled to the hydrolysis of sulfate groups.

Within the free energy landscape characterizing this process the cycle evidently runs along a closed trajectory passing the states *S*1 and *S*2 (Fig. 3F). The transition *S*1 → *S*2 exhibits a negative change in Gibbs free energy (${\rm{\Delta }}{G}_{S1\to S2}^{0}\approx -\,20\,{{\rm{kJmol}}}^{-1}$) and can therefore take place spontaneously. In our experiments however, Sulf1HD required an additional external stimulus (force) to cross the transition stage between *S*1 and *S*2. For Sulf1HD alone the allosteric regulation seems to be hampered. It nevertheless appears well-conceivable, that within the full length protein containing CAT-D, the interstate activation barrier can be surpassed by thermal activation. As mentioned before, this transition goes along with a conformational change into a mechanically strained state that causes Sulf1HD to establish supplementary bonds with the HS chain. In contrast, the transition from *S*2 back to *S*1 is endergonic and requires at least 20 kJmol^−1^. Mere thermal activation appears unlikely as it would hinder the catalytic desulfation process. However, HS desulfation itself releases an adequate amount of energy that would allow the complex to surpass the transition state. We estimated the difference in Gibbs free energy between a sulfated and desulfated HS disaccharide unit according to equation 5 yielding a release of 51.6 kJmol^−1^ per 6-O-sulfate. Therefore, hydrolysis of a single sulfate group is adequate to drive the system across the transition state. In the picture of the inch-worm model the release of a sulfate group leads to partial detaching of Sulf1HD from the HS strand. The mechanical tension built up in the transition *S*1 → *S*2 releases and Sulf1HD can bind to the next downstream binding site. When bound in the state *S*1 the whole process can repeat.

Notably, apart from Sulf1 there are also other polysaccharide-modifying enzymes that exhibit a processive mechanism. For instance, bifunctional GlcNAc N-deacetylase/N-sulfotransferase (NDST), which catalyzes a key step in the initial heparin/HS biosynthesis, apparently generates extended N-sulfated domains in a processive mode^30^. Furthermore, dermatan sulfate 5C epimerase 1 (DSE) was shown to be a processive enzyme which analogously to Sulf1 contains highly charged protein regions (residues E100-D128, D516-Q548 and Q782-858)^31^. However, to our best knowledge none of these systems has been studied by SMFS so far in order to characterize their dissociation behavior and to estimate the free energy landscape.

## Conclusion

In this study we explored the free energy landscape of the catch bond exhibiting hydrophilic domain of the human cell surface sulfatase Sulf1. We furthermore provide an extensive thermodynamic and kinetic description of Sulf1HD/HS binding. By means of single molecule force spectroscopy experiments at several constant pulling velocities we estimated a set of adhesion energies *W*_*n*_ and by applying Jarzynski’s equality, estimated the corresponding free energy landscape. According to the two state two path model we could sketch a two-well energy landscape with two binding states *S*1 (21.1 kJmol^−1^) and *S*2 (40.9 kJmol^−1^), respectively. In our previous work, using the two state two path model we could only quantify the energy difference between the two binding states. In the present study, we estimated the difference in free energy between the bound and unbound states. In full accordance with our previous results the bound states *S*1 and *S*2 differ by approximately 20 kJmol^−1^ ^22^. On the basis of publications by Seffouh and co-workers we drafted a refined model of the allostery driven desulfation process and merged biophysical and biochemical data to a consistent picture^28^. HS desulfation is evidently a cyclic process which can be described by a closed path within the free energy landscape passing the states *S*1 and *S*2. Presumably, this process runs through more than just the two states discussed here. It is likely that there are additional intermediate states that are either too short-lived to be observed in our experiments or, they are only realized in the full length protein. We nevertheless could identify and characterize two states which are crucial in the processive catalytic action of Sulf1. On its trajectory through the energy landscape Sulf1HD most likely undergoes cyclic conformational transitions. Unfortunately, the structure of Sulf1 is still unknown. Therefore, mechanic models like the inch worm is based on functional biophysical and biochemical data. Furthermore, the Sulf1 proccessive desulfation evidently exhibits remarkable similarities to the ATP digesting motor enzyme myosin. Similar to Sulf1HD/HS, actomyosin dissociates in a catch bond-like fashion and, furthermore, both are driven through hydrolysis of a ligand/substrate^12^. Moreover, taking into account HD’s mutliple binding sites and the assumed cyclic conformational transitions, an inch-worm like motion along the HS backbone appears plausible. Hydrolysis of HS 6-O-sulfates certainly provides a sufficient amount of energy to drive the molecular desulfation machinery. However, whether Sulf1 actually can exert forces or if HS desulfation is a solely thermally driven ratchet-like mechanism is still to be shown. Finally, the Jarzynski equality has proven to be a valuable tool for exploring non-trivial energy landscapes which here allowed us to shed light onto the fine-balanced molecular machinery of catalytic HS desulfation.

## Materials and Methods

### Protein expression and purification

For the investigation of Sulf1HD, a fusion protein of an N-terminal maltose binding protein tag followed by the HD of human Sulf1 (residues K417-K735) (MBP-HD) was produced. Expression of MBP-HD was achieved in *Escherichia coli* Rosetta 2 (DE3) (Merck, Darmstadt, Germany) using a plasmid that is based on pMAL-c5X (New England Biolabs, Frankfurt, Germany) and which has been already described^27^. The protein was purified following an established protocol^22^. Briefly, after expression, harvested cells were resuspended in phosphate buffered saline (PBS, prepared from substances of analytical grade (purity ≥99%) and sterile filtered, pH7.3) on ice and lysed. Cleared supernatant was loaded on an amylose affinitiy column (MBP-Trap HP 5 GE Healthcare, Little Chalfont, UK) using an ÄKTAexplorer chromatography system (GE-Healthcare) at 4 °C. After washing out unbound protein with PBS, pH7.3, bound proteins were eluted in one step to 100% elution buffer (10 mM D-maltose in PBS, pH7.3, Sigma Aldrich, purity ≥95%). Fractions of 2 ml each were collected and analyzed via Bradford assay (Coomassie Plus Protein Assay Reagent, Thermo Fisher Scientific), SDS-Polyacrylamide electrophoresis and subsequent Coomassie Brilliant Blue staining^22^.

### Sample preparation

As substrates we used ultra-flat gold surfaces prepared according to the template stripped gold (TSG) procedure^32^. Both, substrates and cantilevers were incubated in a 1 solution of polyethylene glycol di-*N*-succinimidyl ester disulfide linkers (PEG-NHS-ester disulfide, Polypure AS, Oslo, Norway) in water free dimethyl sulfoxide (DMSO, Purity ≥99.9%, Sigma Aldrich, Steinheim, Germany). HS (HS sodium salt, lyophilized, from porcine intestinal mucosa, Celsus Laboratories, Cincinnati, OH, USA) as well as K5-NS (Iduron, Manchester, United Kingdom) were then fused to the cantilever via readily introduced amino groups in coupling buffer (0.2 NaHCO_3 *pH*8.2). MBP-HD (100 mg mL^−1^) was coupled to the immobilized PEG-NHS-esters in PBS pH7.3 with low surface concentration to present only very few potential binding partners to the GAG-coated tip. Even though, the overall interaction rate drops well below 10%, it significantly suppresses the occurrence of multiple binding events^27^. Incubation times were 1 hour for the PEG-NHS linker and 1.5 hours for HS and MBP-HD coupling, respectively. All immobilization procedures were done at room temperature. After incubation, the immobilized substrate surfaces and cantilevers were rinsed extensively with PBS.

### Single molecule force spectroscopy

Single molecule force spectroscopy (SMFS) experiments were conducted at room temperature in PBS buffer at pH7.3 with a commercially available AFM (MFP-3D Bio Asylum Research, Goleta, CA, USA). As force probes we used soft gold-coated silicon nitride force probes with nominal force constants of *k* = 0.03 Nm^−1^ (Olympus Biolever BL-RC150, Tokyo, Japan). Nevertheless, prior to each measurement the cantilevers were calibrated using the thermal fluctuation method with an absolute uncertainty of approx. 10%. The approach velocity and surface dwell time for all experiments were set to 3000 nm s^−1^ and 0.5 s, respectively. The pulling velocities ranged from 20 nm s^−1^ to 3500 nm s^−1^. In a series of control experiments we quantified the unspecific interaction between heparin which exhibits a higher degree of sulfation and therefore bears a stronger specific charge against unmodified and MBP-covered gold substrates^27^. The estimated interaction rates where substantially lower than those acquired for HD and HS. Therefore, SMFS data sets of can be considered as virtually free of unspecific adhesion.

### Data analysis

Jarzyski’s non-equilibrium work theorem allowed us to calculate the Gibbs free energy Δ*G*^0^ directly from a series of non-equlibrium experiments no matter how far from equilibrium the system was probed^33–35^ To estimate Δ*G*^0^ we used Jarzynski’s equality:

1 $$\exp (\,-\,\frac{{\rm{\Delta }}{G}^{0}}{{k}_{{\rm{B}}}T})={\langle \exp (\frac{{W}_{n}}{{k}_{{\rm{B}}}T})\rangle }_{n}.$$

Here *W*_*n*_ and *k*_*B*_*T* depict the work that is dissipated to break an individual bond (*W*_*n*_ < 0) and the thermal energy (*T* = 292 K), respectively. As molecular unbinding is of stochastic nature dissociation events exhibit characteristic spectra of adhesion energies. Though, these spectra are biased by effects such as linker unfolding which adds up to the work that has to be spent to break the bond. It is therefore preferential to probe the system bidirectionally i.e. the forward and backward force extension curve as it is done in (un-) folding experiments of RNA^25,36,37^. However, this is not possible in all types of single molecule dissociation experiments. Therefore, in order to minimize the impact of linker unfolding and to get a fast converging estimate of Δ*G*^0^ one can either reduce the interaction length or sample a ludicrously high number of dissociation events^25,26^. In our experiments, we chose a short linker which only bears 7 PEG units. The length of HS though is polydisperse and can be up to 100 nm. As a result the estimated work for dissociation events with a large interaction lengths are successively biased by the unfolding of the polymer chain. Still, we preferentially observed dissociation events that exhibited an interaction length below 20 nm. For the analysis of the force extension curves, we used a custom-made MATLAB software to validate and analyze the characteristic nonlinear force ramps. Briefly, the force distance curves were corrected for the molecular extension and possible position and deflection offsets. A worm-like chain (WLC) fit is applied to identify specific dissociation events. For evaluating the work and keeping the bias of linker unfolding as low as possible, we only accepted force extension curves that exhibited a contour length in the range of 8 nm to 20 nm (Fig. 3B). Subsequently, we estimated a set of *W*_*n*_ by determining the area under the force profiles^33,34^. Finally, *W*_*n*_ is weighed by applying the Jarzynski equality and solved for Δ*G*^0^. Furthermore, to complement the free energy landscape, the activation energy of the dissociation path $\Delta {G}_{-}^{\ddagger }$ can be determined by means of the Arrhenius equation^38–40^

2 $${k}_{-}^{0}(T)=\nu \exp (\,-\,\frac{{\rm{\Delta }}{G}_{-}^{\ddagger }}{{N}_{A}{k}_{B}T}).$$

where N_*A*_ is the Avogadro constant and $\nu =\tfrac{{k}_{B}T}{h}=6d-12{s}^{-1}$ is the attempt frequency for the crossing of the transition state, respectively. Accordingly, the activation energy of association is estimated as:

3 $${\rm{\Delta }}{G}_{+}^{\ddagger }={\rm{\Delta }}{G}_{-}^{\ddagger }-{\rm{\Delta }}{G}^{0}\mathrm{.}$$

The equilibrium constant *K* and the association rate constant ${k}_{+}^{0}$ in thermodynamic equilibrium are calculated as:

4 $$K=\exp (\,-\,\frac{{\rm{\Delta }}{G}^{0}}{{N}_{{\rm{A}}}{k}_{{\rm{B}}}T})=\frac{{k}_{-}^{0}}{{k}_{+}^{0}}.$$

Finally, we estimated the amount of energy that is released by hydrolysis of a single 6-O-sulfate.

5 $${\rm{\Delta }}G={\rm{\Delta }}G{{}^{{\rm{^{\prime} }}}}^{0}+{N}_{{\rm{A}}}{k}_{{\rm{B}}}T\,{\rm{l}}{\rm{n}}(\frac{[{{\rm{S}}{\rm{O}}}_{4}^{2-}][{\rm{U}}{\rm{A}}-{\rm{G}}{\rm{l}}{\rm{c}}{\rm{N}}{\rm{A}}{\rm{c}}]}{[{\rm{U}}{\rm{A}}-{\rm{G}}{\rm{l}}{\rm{c}}{\rm{N}}{\rm{A}}{\rm{c}}(6{\rm{S}})]{c}^{\circ }})$$

Here, the difference in standard Gibbs free energy in thermal equilibrium at *pH*7 is estimated as *G*′^0^ = 37.2 kJmol^−1^ based on the value for the desulfation of methyl sulfate^41^. The concentration of inorganic sulfate in the interstitial fluid is the same as in blood plasma which was measured to be approx. $[S{O}_{4}^{2-}]=0.4\,{\rm{mM}}$^42,43^. UA-GlcNAc represents the most abundand type of HS disaccharide unit consisting of iduronic acid or glucuronic acid, 1-4-linked to N-acetylglucosamine. UA-GlcNAc(6S) is 6-sulfated UA-GlcNAc (Fig. 2A). The ratio of 6-desulfated to 6-sulfated HS disaccharide units at the cell surface was calculated as [UA − GlcNAc]/[UA − GlcNAc(6S)] = 7.55 and *c*  denotes the unit of concentration^44^.

## Data Availability

All relevant materials, methods and data are within the paper.
